# Post-treatment Recovery in Orbital Arteriovenous Fistulas: A Systematic Review

**DOI:** 10.7759/cureus.75282

**Published:** 2024-12-07

**Authors:** Saif A Badran, Aous Mohammad Qasim, Bashar Ayad Saeed, Mohammed Thakir Ismail, Mohammed Ali Taher, Ahmed A Al-Juboori

**Affiliations:** 1 Department of Surgery, Ibn Sina University of Medical and Pharmaceutical Sciences, Baghdad, IRQ; 2 Department of Surgery, College of Medicine, Ninevah University, Mosul, IRQ; 3 Department of Neurosurgery, Dr. Sa’ad AL-Witri Hospital for Neurosciences, Baghdad, IRQ

**Keywords:** arteriovenous fistula, endovascular treatment, orbital avf, proptosis, transvenous embolization

## Abstract

Orbital arteriovenous fistulas (AVFs) are rare vascular malformations that can cause severe ocular complications. This review evaluates the effectiveness of treatment strategies, focusing on post-treatment recovery and recurrence. A systematic review was conducted using PubMed and Scopus with no date restrictions. Studies were screened based on predefined criteria, and data on presentation, treatment, and outcomes were extracted. The quality assessment followed the CARE (CAse REport)and ROBINS-I (Risk of Bias In Non-randomised Studies of Interventions) guidelines. Fourteen studies were included. Transvenous embolization was the treatment with the highest success and low complication rates. Conservative treatment resulted in the disappearance of some fistulas; surgical and endovascular therapies were also combined for complex AVFs. Transvenous embolization is the preferred treatment method for orbital AVFs, although conservative management might also be sufficient in selected cases, with a multidisciplinary approach being required for complex fistulas.

## Introduction and background

Orbital arteriovenous fistulas (AVFs) are rare and high-flow vascular malformations characterized by the direct abnormal connection of arteries and veins, bypassing the capillary network. These lesions may present with a variety of ophthalmic symptoms including proptosis, chemosis, loss of visual acuity, and extraocular muscle palsy, so their clinical presentation may be similar to carotid-cavernous fistulas [1,2]. While the exact prevalence of orbital AVFs remains unknown due to their rarity, they are usually classified as a subtype of cranial AVFs, with fewer than 20 reported cases in the literature​ [2].

The orbital vascular system is complex, and AVFs in this region pose a significant diagnostic and therapeutic challenge. The abnormal communication between the ophthalmic artery and its accompanying venous drainage system frequently results in vascular congestion with secondary complications of raised intraocular pressure and potential loss of vision​ [1,3]. This constitutes a challenge in the management of such lesions due to the peculiar anatomy of the orbit, being a confined space with vital structures around it, which usually requires a multidisciplinary approach​ [2].

Traditionally, surgical and endovascular approaches have been performed in the treatment of orbital AVFs. Approaches have included direct surgical ligation, transarterial embolization, and, more recently, transvenous coil embolization​ [3]. Despite these, most series consider the AVF as a difficult malformation to treat, with recurrence remaining one of the major issues, particularly when the lesions are diffuse​ [1]. Newer therapeutic methods against angiogenesis also present a promising new approach​.

This systematic review aims to evaluate the post-treatment recovery of patients with orbital AVFs, focusing on the long-term outcomes and quality of life following various therapeutic interventions. By synthesizing data on recurrence rates, complications, and patient well-being, this review seeks to provide insights into the efficacy of current treatments and highlight the most effective strategies for optimizing patient recovery and minimizing long-term sequelae.

## Review

Method

**Figure 1:**
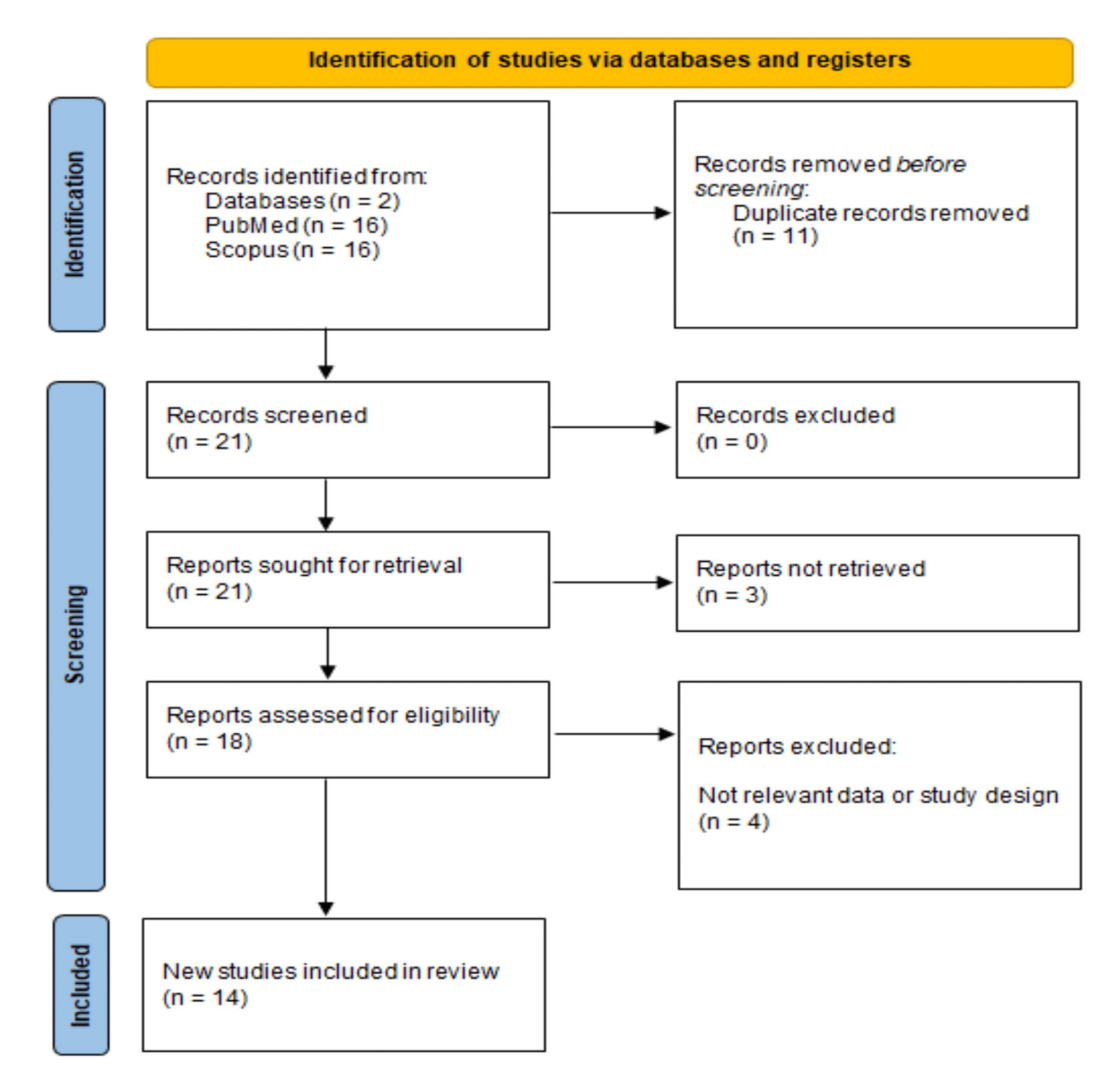
PRISMA flowchart of the included studies PRISMA: Preferred Reporting Items for Systematic Reviews and Meta-Analyses

This systematic review was conducted in accordance with the PRISMA (Preferred Reporting Items for Systematic Reviews and Meta-Analyses) guidelines [4], ensuring rigorous methodology (Figure 1).

The databases PubMed and Scopus were thoroughly searched using the terms "orbital arteriovenous fistula" OR "orbital AVF", with no date restrictions but limiting the results to English-language publications. To assist in managing the large volume of retrieved articles, Rayyan (Cambridge, MA, USA), a tool designed for systematic review management, was employed. After removing duplicates, the initial screening of titles and abstracts was carried out.

Eligibility Criteria and Study Selection

We included studies that examined patients with orbital AVFs, encompassing both traumatic and spontaneous cases. Studies needed to provide data on post-treatment recovery, recurrence rates, and long-term patient outcomes. Eligible articles included case reports, case series, and cohort studies. Two independent reviewers screened all retrieved articles. Titles and abstracts were first reviewed for relevance. Full-text articles were then obtained for those meeting the initial criteria, and their inclusion was confirmed based on predetermined eligibility. Any discrepancies between reviewers were resolved through discussion or, when necessary, consultation with a third reviewer.

Data Extraction

A structured data extraction form was used to gather the following information from each included study: study design, country, sample size, population demographics, clinical presentation, diagnostic methods, treatment approaches, and outcomes. In addition, data on complications and follow-up duration were recorded. The extraction was performed by two reviewers independently, ensuring consistency and accuracy. Any disagreements were resolved through consensus.

Quality Assessment

Each included study was evaluated for quality using the CARE (CAse REport) guidelines for case reports [5] and the ROBINS-I (Risk of Bias In Non-randomised Studies of Interventions) tool for cohort studies [6]. The risk of bias was carefully assessed in areas such as study selection, reporting bias, and follow-up periods. Studies with minimal bias and high methodological rigor were prioritized in the analysis.

Synthesis of Results

Due to the variability in study designs and reported outcomes, a narrative synthesis approach was chosen. Key outcomes, such as recurrence rates, post-treatment complications, and improvements in clinical symptoms (e.g., proptosis reduction, visual acuity), were analyzed. Studies were grouped based on treatment modality (e.g., surgical vs. endovascular), and outcomes were compared across these categories to draw meaningful conclusions about the efficacy of different interventions.

Results

**Figure 2:**
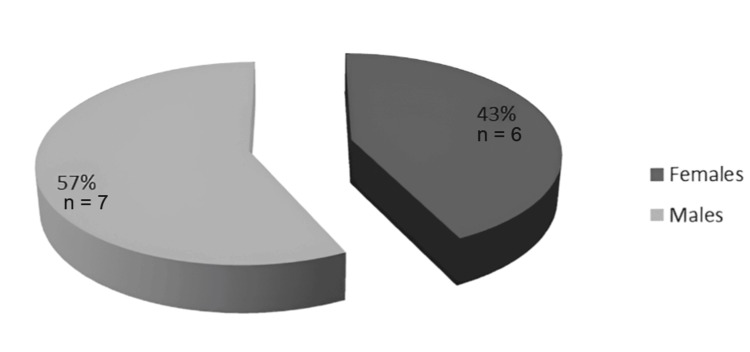
Gender distribution in orbital arteriovenous fistula studies

This systematic review assessed 14 studies involving patients with orbital AVFs, focusing on clinical presentation, diagnostic strategies, treatment modalities, and post-treatment outcomes (Figure 2).

**Figure 3:**
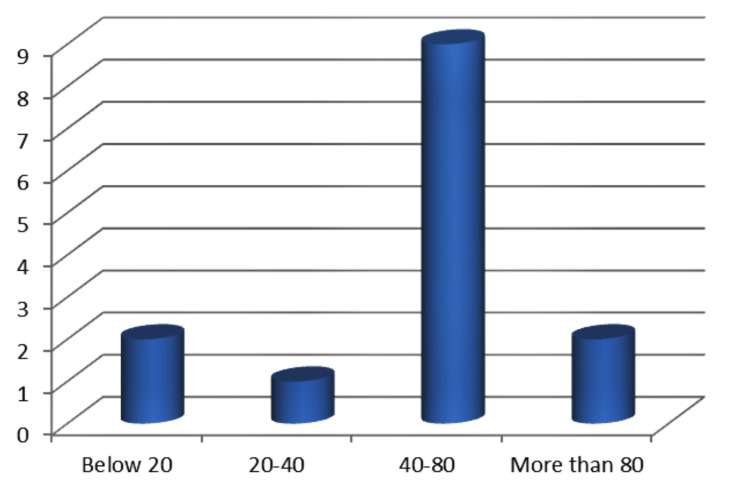
Age distribution of patients in orbital arteriovenous fistula studies References: [7,8,10-20]

The studies included both traumatic and spontaneous cases of orbital AVFs, reflecting the diverse nature of this condition (Table 1, Figure 3) [7-20].

**Table 1:** Summary of case characteristics, diagnostic approaches, and outcomes in orbital arteriovenous fistula patients AVF: arteriovenous fistula, CBCT: cone-beam computed tomography, CT: computed tomography, DSA: digital subtraction angiography, IOphV: inferior ophthalmic vein, IOV: inferior ophthalmic vein, LVM: large vascular malformation, MVA: motor vehicle accident, OCT: optical coherence tomography, OCTA: optical coherence tomography angiography, SOV: superior ophthalmic vein, VEGF: vascular endothelial growth factor

Study ID	Study reference	Study design	Country	Sample size	Population characteristics	Laterality	Clinical presentation	Diagnosis methods	Treatment approaches	Outcomes	Complications	Follow-up duration
1	Rush et al., 1983 [7]	Case report	USA	1	63-year-old male with head trauma	Left	On the following day, the patient experienced pain, proptosis, and visual loss. An ocular examination revealed a blind, immobile, and proptotic left eye. The patient presented with left blepharoptosis, chemosis, 25% hyphema, and a dense lens opacity that occluded the posterior pole.	An orbital CT scan revealed a well-defined soft tissue mass in the left orbital apex, consistent with a retrobulbar hematoma, along with a massively enlarged left superior ophthalmic vein. Laboratory tests showed reactive serum and spinal fluid FTA-abs. A selective left internal carotid arteriogram indicated minimal enlargement of the initial segment of the left ophthalmic artery, which was connected to an aneurysmally dilated vessel draining into the enlarged superior ophthalmic vein. Additionally, a 2 mm aneurysm was identified at the origin of the left ophthalmic artery.	Ligation of the intracranial left ophthalmic artery	Reduced proptosis and chemosis, but the left eye remained blind	None reported	10 days
2	Fourman et al., 1989 [8]	Case report	USA	2	Case 1: 17-year-old female, history of chronic glomerulonephritis Case 2: 73-year-old female, history of bilateral sixth nerve palsy	Case 1: Right Case 2: Right	Case 1: Right-sided proptosis, increased intraocular pressure, chemosis, and shallow anterior chamber. Case 2: Right-sided conjunctival engorgement, diplopia, shallow anterior chamber, increased intraocular pressure, bilateral sixth nerve palsy.	Both cases diagnosed with arteriogram: demonstrated the presence of arteriovenous fistulas. Case 1 also utilized gonioscopy and ophthalmoscopy for assessment.	Case 1: Surgical closure of the orbital fistula. Case 2: Embolization of the dural arteriovenous fistula, followed by topical treatments (timolol, pilocarpine) and laser iridotomy.	Case 1: Reduction of intraocular pressure and resolution of ciliochoroidal detachment; visual acuity improved from 20/80 to 20/200 post-surgery. Case 2: Resolution of visual symptoms and intraocular pressure normalized; maintained good vision (10 mm Hg intraocular pressure) one year later.	None reported in either case	Case 1: Six months Case 2: One year
3	Keizer et al., 2003 [9]	Case series	Netherlands	68	Various patients; included traumatic and spontaneous cases	Mixed	The most common symptom was exophthalmos, observed in 65 patients (96%), indicating significant proptosis due to increased venous pressure. Specific epibulbar loops were noted in 66 cases (97%), while motility disturbances and glaucoma were present in 45 patients (66%). Other symptoms included murmurs (40%), pulsations (15%), ocular or orbital pain (16%), and eyelid swelling (13%). Additionally, 26 patients (38%) experienced decreased visual acuity, often requiring treatment. Definitive visual impairment caused by CCF was noted in six cases (9%), and orbital fissure syndrome occurred in eight cases (12%).	Diagnosed using angiography: 101 cases confirmed CCF presence - MRI and Doppler imaging for hemodynamic assessment	Treatment methods varied: Conservative management (58% success), balloon embolization (94.5% success), direct surgery in severe cases	- Successful management in 39 cases with proptosis reduction. - 17 out of 18 embolizations successful. - 82% of conservatively treated dural fistulas improved.	Complications included progressive visual impairment in traumatic cases. - Some cases resulted in death (due to vascular complications).	Followed up for various durations; chronic cases often monitored for more than a year
4	Deguchi et al., 2005 [10]	Case report	Japan	1	51-year-old male whose left visual acuity had been impaired since childhood	Left	By 2000, he experienced worsening visual acuity, along with left-sided exophthalmos and chemosis, prompting his referral to an ophthalmologist. Upon examination, he demonstrated exophthalmos of 9.5 mm in the left eye, with visual acuity reduced to less than 20/800, allowing only for finger counting at 10 cm. A Goldmann perimetry examination revealed a substantial visual defect in the left central visual field.	Left internal carotid artery angiography, which identified an arteriovenous shunt between the ophthalmic artery and the inferior orbital vein. Notably, the blood flow from the inferior orbital vein did not drain into the cavernous sinus but refluxed into the superior orbital vein, draining into the facial vein via the angular vein. Additionally, superselective ophthalmic artery angiography demonstrated no nidus typical of an arteriovenous malformation (AVM), allowing for the identification of the arteriovenous fistula around the optic nerve sheath.	The patient underwent two transvenous embolization procedures to treat the purely intraorbital arteriovenous fistula. Initially, local anesthesia was administered without systemic heparinization. A no. 6 French guiding catheter was inserted through the right femoral vein and advanced to the left jugular vein. Using a Rapid Transit microcatheter, the inferior ophthalmic vein (IOphV) was accessed and embolized with mechanically detachable coils and fiber-covered platinum coils. Although the embolization was intended to occlude the fistula, the intervention was terminated when the arteriovenous shunt appeared to have resolved. Three months later, the patient experienced a recurrence of chemosis and exophthalmos, prompting a second embolization procedure. Again, local anesthesia was used, and a guiding catheter was positioned in the left jugular vein to facilitate the procedure.	Following the initial transvenous embolization, the patient experienced a partial resolution of symptoms, but three months later, there was a recurrence of chemosis and exophthalmos. This necessitated a second embolization procedure. After the second procedure, the patient's symptoms improved significantly. The exophthalmos was reduced, with measurements indicating that the left eye's protrusion had decreased. Additionally, visual acuity in the left eye improved to 20/100, and a Goldmann perimetry examination showed a marked improvement in the left central visual field defect.	Despite the successful outcome, the patient faced complications related to the embolization, including transient postoperative discomfort. However, these complications were managed effectively, allowing for a satisfactory recovery.	Six months
5	Frankefort et al., 2005 [11]	Case report	Belgium	1	84-year-old female with Alzheimer’s disease	Left	Presenting symptoms: left-sided redness, conjunctival chemosis, proptosis, and elevated intraocular pressure (40 mmHg). Her best corrected visual acuity was 3/10 in the right eye and 2/10 in the left eye.	Diagnosis confirmed via carotid angiography: Revealed shunt between the right internal carotid artery branches and left orbital vein.	Conservative management with corticosteroids, intravenous acetazolamide, and topical timolol.	Improvement in intraocular pressure, normalization of symptoms; visual acuity improved to 5/10; mild conjunctival redness persisted.	No complications reported during hospitalization.	Two months post-treatment
6	Subramanian et al., 2005 [12]	Case report	USA	1	33-year-old male with diplopia and pain in the right eye	Right	Presenting symptoms: redness, pain, diplopia, and mild proptosis in the right eye; normal visual acuity and color vision.	Magnetic resonance imaging and digital subtraction angiography identified a vascular lesion in the inferior aspect of the right orbit, revealing an orbital arteriovenous fistula (AVF) between the ophthalmic artery and inferior ophthalmic vein (IOV). Blood flow from the IOV drained into the superior ophthalmic vein and then into the cavernous sinus and inferior petrosal sinus.	Transvenous embolization of the AVF using Onyx® and thrombogenic coils via the right facial vein.	Complete resolution of symptoms; visual acuity improved to 20/20; no residual flow seen in the follow-up DSA three months post-embolization.	Initial worsening of lid edema and chemosis post-embolization; resolved within two days.	One month post-embolization
7	Mishra et al., 2013 [13]	Case report	India	1	50-year-old female with a history of proptosis and conjunctival chemosis	Right	Symptoms included 18 months of right eye proptosis, upper lid swelling, conjunctival chemosis, recent retro-orbital pain, and decreased vision.	Diagnosis via CT scan: revealed thrombosed varicose superior ophthalmic vein and intraorbital AVF. B-scan ultrasound with color Doppler: Confirmed diagnosis without catheter angiography.	Direct surgical excision of thrombosed SOV via a fronto-orbital approach.	Complete resolution of symptoms; visual acuity improved and proptosis decreased post-surgery; CT confirmed decompression of the orbit.	No significant complications reported; successful surgical outcome.	Six months post-surgery
8	Lee et., 2017 [14]	Case report	USA	1	82-year-old female with a history of bilateral chronic eye pain and mild trauma	Right	Presenting symptoms: Right-sided proptosis, chemosis, eyelid edema, and blurred vision; history of MVA with airbag deployment.	Diagnosis confirmed via CT Angiography: Revealed an intraorbital inferior ophthalmic vein AVF. Six-vessel cerebral angiography: Confirmed the AVF and venous drainage patterns.	Transvenous embolization of the AVF using Onyx® and thrombogenic coils via the right facial vein.	Complete resolution of symptoms; visual acuity improved to 20/20; no residual flow of the AVF at the follow-up angiography.	Initial worsening of lid edema and chemosis post-embolization; resolved within two days.	One month post-embolization
9	Sato et al., 2017 [15]	Case report	Japan	1	55-year-old male with a history of right eye symptoms post-MVA	Right	Symptoms included right exophthalmos, chemosis, diplopia, and reduced vision; a history of gradual worsening over four months.	Magnetic resonance (MR) imaging and MR angiography indicated a dilated superior orbital vein (SOV), suggestive of a cavernous sinus dural AVF. However, diagnostic cerebral angiography confirmed an AVF in the right orbit fed by a branch of the right ophthalmic artery, draining into the right facial vein via the SOV and inferior ophthalmic vein (IOV). High-resolution cone-beam computed tomography (CBCT) further clarified the shunt point and detailed anatomy of the orbital vessels, showing the AVF surrounding the optic nerve.	Transvenous embolization via the inferior orbital vein using platinum coils and Onyx® embolization material. Due to the high risk of trans-arterial embolization, a transvenous approach was planned for treatment.	Complete resolution of symptoms; visual acuity improved post-embolization; follow-up imaging showed obliteration of the AVF.	Initial worsening of lid edema and chemosis post-embolization, which resolved within two days.	One month post-embolization
10	Putthirangsiwong et al., 2018 [16]	Case report	Thailand	1	11-year-old male	Right	Symptoms included acute proptosis, sudden visual loss, and marked chemosis; presented after showering.	Diagnosis confirmed via CT scan: Revealed a large right intraconal lesion with internal hemorrhage. Carotid angiography: Confirmed AVF and associated vascular anatomy.	Transvenous embolization of the AVF using coils and N-butyl cyanoacrylate glue, combined with direct injection into the LVM and subsequent surgical excision.	Good clinical response; significant decrease in proptosis and improvement in visual acuity; no recurrence at two-year follow-up.	No significant complications reported; successful surgical outcome.	Two years post-treatment
11	Thomas et al., 2018 [17]	Case report	USA	1	69-year-old female, referred for evaluation of macular degeneration	Right	Symptoms included right eye choroidal pulsations, normal visual acuity, and a history of mild visual symptoms; no prior trauma.	Diagnosis confirmed via optical coherence tomography (OCT): Noted choroidal pulsations. Indocyanine green angiography: Revealed engorgement of choroidal vasculature. OCTA: Demonstrated vascular changes.	Anti-VEGF therapy initiated for mild stasis retinopathy; endovascular treatment considered if symptoms progressed.	Stabilization of visual symptoms; choroidal pulsations noted on OCT without significant deterioration; no further visual deficits at follow-up.	Mild stasis retinopathy treated with anti-VEGF; no major complications reported.	Follow-up for six months post-treatment
12	Tsutsumi et al., 2019 [18]	Case report	Japan	1	73-year-old male with progressive proptosis for one month.	Right	Symptoms included considerable proptosis, hyperemia in the lower eyelid, chemosis, and total ophthalmoplegia on the right side.	Diagnosis via CT and MRI: Revealed a round retro-orbital mass and irregular lesion in the orbital apex. Cerebral angiography: Confirmed AVF fed by maxillary and ophthalmic arteries, draining into the inferior ophthalmic vein.	Transvenous coil embolization of the AVF via facial and superior ophthalmic veins, followed by microsurgical resection of the hemangioma two months later.	Complete resolution of proptosis and ophthalmoplegia; histological diagnosis confirmed arteriovenous hemangioma post-resection.	No complications reported; satisfactory surgical outcomes.	Two months post-surgery
13	Akamatsu et al., 2020 [19]	Case report	Japan	1	53-year-old male with an aneurysm-like lesion compressing the left optic chiasm	Left	Symptoms included proptosis, visual disturbances, and a significant mass compressing the optic chiasm; patient presented with gradual worsening over weeks.	Diagnosis via CT angiography: Demonstrated an intraorbital AVF and associated abnormal vessels. MRI: Confirmed the mass effect on the optic chiasm.	Combined surgical and endovascular treatment: Translocating the basal vein of Rosenthal to facilitate direct catheterization for embolization, followed by coil embolization of the AVF.	Complete obliteration of the AVF confirmed by follow-up imaging; significant symptom resolution; no visual deficits reported.	No complications reported; successful surgical and endovascular treatment.	One year post-treatment
14	Hirano et al., 2023 [20]	Case report	Japan	1	70-year-old male with no history of trauma was aware of the deterioration of his visual acuity 6 months before his visit.	Left	Symptoms included gradual visual acuity decline over six months without proptosis or chemosis; initial visual acuity measured at 0.3.	Diagnosis confirmed via MRI: Marked dilation of the left SOV. Cerebral angiography: Revealed AVF supplied by the ophthalmic artery draining into the SOV.	Transvenous embolization of the AVF via the facial vein, using coils to reduce venous pressure on the optic nerve.	Marked improvement in visual acuity from 0.3 to 0.9 by postoperative day 7; significant reduction in the SOV on follow-up imaging.	No complications reported; the procedure was well-tolerated.	Three months post-treatment

The overall quality of the papers included in this review was generally high, with most studies adhering to well-established reporting standards (Tables 2-3) [7-20].

**Table 2:** Quality assessment of case reports on orbital AVFs using CARE guidelines AVF: arteriovenous fistula, CARE: CAse REport

Study ID	First author	Patient information	Clinical findings	Diagnostic assessment	Therapeutic interventions	Follow-up outcome	Discussion conclusions	Overall quality
1	Rush et al. (1983) [7]	Comprehensive	Detailed	Thorough	Well-documented	Reported	Relevant	High
2	Fourman et al. (1989) [8]	Comprehensive	Detailed	Thorough	Well-documented	Reported	Relevant	High
3	Deguchi et al. (2005) [10]	Comprehensive	Detailed	Thorough	Well-documented	Reported	Relevant	High
4	Frankefort et al. (2005) [11]	Comprehensive	Detailed	Thorough	Well-documented	Reported	Relevant	High
5	Subramanian et al. (2005) [12]	Comprehensive	Detailed	Thorough	Well-documented	Reported	Relevant	High
6	Mishra et al. (2013) [13]	Comprehensive	Detailed	Thorough	Well-documented	Reported	Relevant	High
7	Lee et al. (2017) [14]	Comprehensive	Detailed	Thorough	Well-documented	Reported	Relevant	High
8	Sato et al. (2017) [15]	Comprehensive	Detailed	Thorough	Well-documented	Reported	Relevant	High
9	Putthirangsiwong et al. (2018) [16]	Comprehensive	Detailed	Thorough	Well-documented	Reported	Relevant	High
10	Thomas et al. (2018) [17]	Comprehensive	Detailed	Thorough	Well-documented	Reported	Relevant	High
11	Tsutsumi et al. (2019) [18]	Comprehensive	Detailed	Thorough	Well-documented	Reported	Relevant	High
12	Akamatsu et al. (2020) [19]	Comprehensive	Detailed	Thorough	Well-documented	Reported	Relevant	High
13	Hirano et al. (2023) [20]	Comprehensive	Detailed	Thorough	Well-documented	Reported	Relevant	High

**Table 3:** ROBINS-I assessment of the included studies ROBINS-I: Risk of Bias In Non-randomised Studies of Interventions

Study ID	Authors	Confounding	Selection of patients	Classification of interventions	Deviations from intended interventions	Missing data	Measurement of outcomes	Selection of reported results	Overall risk of bias
1	Keizer et al. (2003) [9]	Moderate	Low	Moderate	Low	Low	Moderate	Moderate	Moderate

Clinical Presentation

Proptosis and chemosis were the most consistently reported symptoms across studies, often indicating significant venous pressure within the orbit. Keizer et al. (2003) [9] reported that 96% of patients exhibited exophthalmos, a hallmark of orbital AVFs. Other common symptoms included ocular pain, diplopia, and glaucoma, particularly in chronic cases, while visual acuity was often impaired in severe presentations​. Interestingly, some cases presented without the classic signs of proptosis, such as the 70-year-old male described by Hirano et al. (2023) [20], who primarily experienced gradual visual acuity decline due to venous compression​.

Laterality was predominantly unilateral, with a slight predilection for the right orbit in several cases. For example, in the report by Subramanian et al. (2005) [12], the patient presented with right-sided proptosis and pain​. Bilateral involvement was rare, but cases with complex clinical presentations, such as those involving both motility disturbances and pulsations, indicated more diffuse vascular involvement​.

Diagnostic Modalities

The diagnostic workup of orbital AVFs is heavily reliant on advanced imaging, with CT angiography and digital subtraction angiography (DSA) being the gold standard for identifying arteriovenous connections and assessing hemodynamics. MRI and magnetic resonance angiography (MRA) were used in several studies to evaluate the soft tissue and vascular components in detail. For example, Sato et al. (2017) [15] utilized high-resolution cone-beam CT to pinpoint the AVF location and clarify its anatomical relationship with orbital structures, especially the optic nerve, which played a crucial role in guiding the subsequent transvenous embolization​.

In some cases, non-invasive imaging modalities were employed to support diagnosis. OCT was particularly valuable in detecting vascular changes in patients with subtle presentations. For instance, in Thomas et al. (2018) [17], OCT revealed choroidal pulsations, leading to the diagnosis of an AVF, despite the lack of other classical signs like proptosis​. Color Doppler ultrasonography was also used as a complementary tool, especially in cases where angiography was not readily accessible, as demonstrated by Mishra et al. (2013) [13] in a developing country setting​.

Surgical and Endovascular Approaches

Treatment of orbital AVFs often involves a combination of surgical ligation and endovascular techniques, depending on the complexity of the fistula and the patient’s clinical condition. Transvenous embolization was the most frequently reported intervention, achieving high success rates in terms of symptom resolution and fistula obliteration. This technique involves accessing the fistula via the venous system, typically through the inferior ophthalmic vein (IOV) or superior ophthalmic vein (SOV), and deploying coils or embolic agents such as Onyx® or N-butyl cyanoacrylate glue.

In the study by Deguchi et al. (2005) [10], the patient underwent two transvenous embolization procedures to treat a purely intraorbital AVF. Despite an initial recurrence three months after the first intervention, the second embolization achieved significant improvement in both visual acuity and exophthalmos, with no long-term complications reported​. Similarly, Lee et al. (2017) [14] described a successful transvenous embolization using Onyx® and thrombogenic coils, leading to complete resolution of symptoms and improved visual acuity​.

In more severe or complex cases, direct surgical intervention was necessary. Mishra et al. (2013) [13] reported on a case where direct surgical excision of a thrombosed SOV was performed due to an intraorbital AVF with associated varices. The surgery resulted in complete resolution of symptoms, including proptosis and visual improvement, without significant complications​. Surgical ligation of feeding arteries, as seen in Rush et al. (1983) [7], was another option, although it was less commonly employed in more recent studies due to the preference for minimally invasive techniques​.

A combined surgical and endovascular approach was particularly effective in complex cases. In the study by Akamatsu et al. (2020) [19], a combination of surgical translocation of the basal vein of Rosenthal and coil embolization successfully obliterated the AVF, with complete resolution of symptoms and no recurrence during one-year follow-up​.

Post-treatment Outcomes

**Figure 4:**
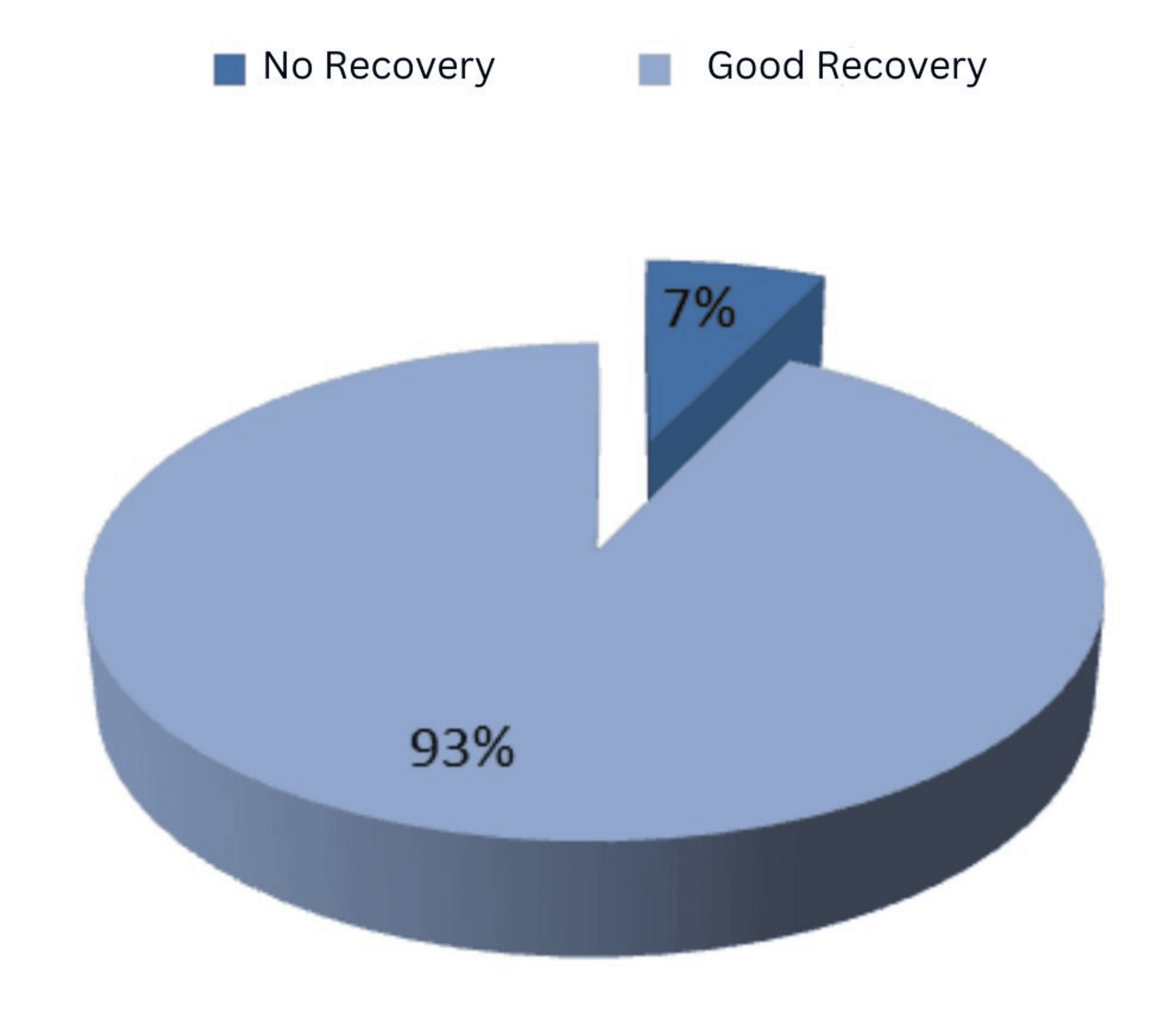
Outcomes of patients in orbital arteriovenous fistula studies References: [7,8,10-20]

Post-treatment recovery was generally favorable, with most patients experiencing significant reductions in proptosis and chemosis (Figure 4).

Keizer et al. (2003) [9] reported that 82% of patients treated with conservative measures showed improvement in symptoms, although those requiring embolization had a higher success rate (94.5%)​. Visual acuity improved in most cases, with several patients regaining near-normal vision. For example, in Subramanian et al. (2005) [12], the patient’s visual acuity improved to 20/20 post-embolization, and no residual flow was observed on follow-up angiography​.

Recurrence was rare but occurred in a few cases, such as in Deguchi et al. (2005) [10], where the patient experienced a recurrence of symptoms three months after the first embolization, necessitating a second procedure​.

Complications

Complications were minimal, with most patients reporting only transient postoperative discomfort, such as lid edema and chemosis, which resolved within a few days. In the rare instances of complications, such as the patient in Frankefort et al. (2005) [11], elevated intraocular pressure was successfully managed conservatively, with no lasting adverse effects​. Overall, the majority of procedures were well-tolerated, and no major complications were reported in the reviewed studies.

Follow-Up and Long-Term Outcomes

The follow-up periods have been anything from one month to over two years, allowing observation of the treatments' long-term durability. Putthirangsiwong et al. (2018) [16] reported no recurrence of symptoms at two-year follow-up, while shorter follow-up periods showed early resolution of symptoms but limited data on long-term recovery​. In the instances where embolization had taken place, the extended follow-up was essential since it would be a potential time for recurrence and therefore observation of sustained efficacy of the intervention.

Discussion

This systematic review synthesizes the current evidence regarding the clinical presentation, diagnostic modalities, treatment options, and long-term outcomes in patients with orbital AVFs. Orbital AVFs are rare vascular malformations that pose significant diagnostic and therapeutic challenges due to their proximity to critical structures and their variable clinical presentations. The findings from this review contribute valuable insights into the effectiveness of various treatment modalities and underscore the need for individualized approaches based on patient characteristics and fistula complexity.

The most common symptoms in the articles reviewed were exophthalmos and chemosis impairing vision. Keizer et al. (2003) [9] mentioned that the proportion of patients with exophthalmos was 96%, which usually directly results from the increased venous pressure inside the orbit​. Interestingly, although proptosis has been a classic sign, in a few cases, there was no classical proptosis, and vision slowly deteriorated due to the compressive effect of the venous system on the optic nerve​, according to the case by Hirano et al. in 2023 [20]. The variability in presentation points to the importance of a broad diagnostic approach in the possible disclosure of AVFs in a patient, especially in those cases where the signs are very minimal or not too characteristic.

Laterality was predominantly unilateral in most cases, with a slight predilection for right-sided involvement. Bilateral AVFs were exceedingly rare, but cases involving more complex presentations, such as motility disturbances or pulsations, suggested more diffuse vascular involvement. The variability in clinical presentation suggests that early recognition and appropriate diagnostic workup are critical to preventing irreversible visual loss and managing orbital AVFs effectively.

Two cases of spontaneous orbital AVFs were reported with similar clinical presentations. Hamada et al. (2006) [21] described a 55-year-old male with a six-month history of right upper lid swelling, conjunctival chemosis, and proptosis without trauma, confirmed by imaging to be an AVF draining into the SOV. Similarly, Ohtsuka and Hashimoto (1999) [22] reported a 73-year-old woman with a one-year history of mild proptosis, upper eyelid swelling, and a faint bruit also diagnosed with an AVF involving dilated superior and inferior ophthalmic veins. Both cases highlight the typical orbital congestion signs seen in spontaneous AVFs​.

The diagnostic evaluation of orbital AVFs relies heavily on advanced imaging techniques. CT angiography and DSA remain the gold standards for identifying arteriovenous connections and assessing hemodynamics. Sato et al. (2017) [15] utilized high-resolution cone-beam CT to precisely identify the shunt location and its anatomical relationship to the optic nerve, which was critical in planning a successful transvenous embolization​. These imaging techniques enable precise localization of AVFs and provide critical information on venous drainage patterns, which guide treatment decisions.

For instance, in some cases, OCT and other non-invasive imaging techniques provided essential evidence of subtle vascular changes. Thomas et al. (2018) [17] employed OCT to evidence a pulsation in the choroid, which ultimately diagnosed an AVF in a case that was otherwise presented with minimum clinical evidence​. Color Doppler ultrasonography also proved useful in resource-limited settings, as was pointed out by Mishra et al. (2013) [13], indicating the use of this modality when advanced imaging is not available​. These results stress the importance of a multimodal imaging approach, especially in cases with atypical presentation.

The treatment of orbital AVFs typically involves either transvenous embolization or direct surgical ligation, depending on the complexity of the fistula and the patient’s overall clinical condition. Transvenous embolization, often using materials such as Onyx® or platinum coils, was the most frequently employed intervention across studies. This approach consistently achieved high success rates, with complete resolution of symptoms in most cases​. For example, Subramanian et al. (2005) [12] reported a case where transvenous embolization via the facial vein led to the complete resolution of symptoms, with visual acuity improving to 20/20 post-procedure​. Similarly, Deguchi et al. (2005) [10] demonstrated that repeated transvenous embolization successfully controlled a recurring AVF, with marked improvements in both exophthalmos and visual acuity​. In more severe cases, or when embolization alone was insufficient, a combined approach involving both surgical and endovascular techniques was used. Akamatsu et al. (2020) [19] described a complex case in which translocation of the basal vein of Rosenthal combined with coil embolization successfully obliterated the AVF, resulting in a complete resolution of symptoms without recurrence​. This hybrid approach highlights the necessity of tailoring treatment to the anatomical and hemodynamic complexities of individual cases.

In the paper by Cheng et al. (2009) [23], one 50-year-old patient with intraorbital AVFs was not intervened due to technical difficulty and high risk associated with vascular treatment. The patient was managed only with conservative management on regular follow-up; spontaneous resolution of the fistula within six months was observed along with remarkable improvement in visual acuity with the resolution of proptosis. On the other hand, Naqvi et al. (2013) [24] reported a case of a 72-year-old patient presenting with progressive proptosis that was successfully treated by way of transvenous embolization after the direct surgical exposure of the SOV. Safe embolization in this case could be performed without compromise of the central retinal artery and the symptoms completely resolved within one month.

The overall post-treatment outcomes for patients with orbital AVFs were favorable. Keizer et al. (2003) [9] reported that 94.5% of patients who underwent embolization experienced a significant reduction in proptosis and symptom resolution​. Visual acuity improvements were commonly reported, with some patients, such as those treated by Subramanian et al. (2005) [12], achieving full visual recovery post-embolization​. However, recurrence of symptoms was reported in a few cases, such as the patient treated by Deguchi et al. (2005) [10], who required a second embolization procedure to fully control the AVF. Despite the generally positive outcomes, there were instances of transient postoperative complications, such as lid edema and chemosis, which resolved within days. Major complications were rare, and most procedures were well-tolerated, reinforcing the safety and efficacy of both embolization and surgical approaches​.

In Yazici et al. (2007) [25], a 13-year-old girl developed an intraorbital AVF following a penetrating injury. The fistula was between the ophthalmic artery and the SOV. Although an attempted embolization through the femoral vein failed and led to SOV thrombosis, the condition resolved spontaneously within a month. The patient’s vision improved, and there was no recurrence during the 23-month follow-up.

Although the studies included in this review provided valuable insights into the management of orbital AVFs, several limitations were noted. Many of the included studies were case reports or small case series, which limited the generalizability of the findings. Furthermore, there was significant variability in follow-up durations, with some studies providing only short-term outcome data from few days to few months. Longer-term follow-up is critical to fully understand the durability of treatments and the risk of recurrence. Future studies should aim to include larger cohorts and longer follow-up periods to better assess the long-term efficacy of various treatment modalities.

Moreover, newer modes of therapy also include the use of antiangiogenic agents. These newer modes of treatment are emerging and have shown their promise in the management of complicated or recurrent AVFs. There is a possibility that the future may combine such therapies with classic endovascular techniques.

## Conclusions

Orbital AVFs are uncommon vascular malformations that require prompt diagnosis and timely intervention. This review places special emphasis on the efficacy of the mainstay of treatment - transvenous embolization - with very good symptomatic improvement and very low recurrence rates. Although spontaneous resolution can occur with conservative management in selected cases, complicated AVFs do better when managed with combined surgical and endovascular approaches. Improved diagnostic imaging has greatly enhanced diagnosis and treatment planning. Future research should focus on enhancing treatment protocols and exploring novel therapies to optimize patient outcomes.
